# Regen med therapeutic opportunities for fighting COVID‐19

**DOI:** 10.1002/sctm.20-0245

**Published:** 2020-08-27

**Authors:** Anthony Atala, Alicia Henn, Martha Lundberg, Taby Ahsan, Jordan Greenberg, Jeff Krukin, Steven Lynum, Cat Lutz, Kyle Cetrulo, Mohammad Albanna, Taciana Pereira, Shannon Eaker, Joshua Hunsberger

**Affiliations:** ^1^ Wake Forest Institute for Regenerative Medicine Winston‐Salem North Carolina USA; ^2^ Biospherix Lacona, New York USA; ^3^ National Heart, Lung and Blood Institute (NHLBI) Bethesda Maryland USA; ^4^ RoosterBio Frederick Maryland USA; ^5^ Organabio Miami, Florida USA; ^6^ Orbital Transports Chicago Illinois USA; ^7^ PHC Group Livingston New Jersey USA; ^8^ Jackson Labs Mount Desert Island, Maine USA; ^9^ International Perinatal Stem Cell Society, Inc. Westport Connecticut USA; ^10^ Humabiologics, Inc. Phoenix Arizona USA; ^11^ Allevi Philadelphia Pennsylvania USA; ^12^ Cytiva Marlborough Massachusetts USA; ^13^ Regenerative Medicine Manufacturing Society Winston‐Salem North Carolina USA

**Keywords:** animal models, COVID‐19, mesenchymal stromal/stem cells, organoids, regenerative medicine, SARS‐CoV‐2

## Abstract

This perspective from a Regenerative Medicine Manufacturing Society working group highlights regenerative medicine therapeutic opportunities for fighting COVID‐19. This article addresses why SARS‐CoV‐2 is so different from other viruses and how regenerative medicine is poised to deliver new therapeutic opportunities to battle COVID‐19. We describe animal models that depict the mechanism of action for COVID‐19 and that may help identify new treatments. Additionally, organoid platforms that can recapitulate some of the physiological properties of human organ systems, such as the lungs and the heart, are discussed as potential platforms that may prove useful in rapidly screening new drugs and identifying at‐risk patients. This article critically evaluates some of the promising regenerative medicine‐based therapies for treating COVID‐19 and presents some of the collective technologies and resources that the scientific community currently has available to confront this pandemic.

**Significance statement**

Regenerative medicine is uniquely positioned to provide advanced organoid models to understand the infection mechanism of, identify patients at risk for, and develop ways to prevent COVID‐19, as well as to introduce innovative treatments that have immunomodulatory and regenerative properties. The Regenerative Medicine Manufacturing Society (RMMS) and *STEM CELLS Translational Medicine* (SCTM) collaborated to create a platform on the STEM CELLS Portal (https://stemcellsportal.com/regenerative-medicine-covid-19-resources) for sharing regenerative medicine resources to address the COVID‐19 pandemic in three areas: models, cell therapies, and technologies. This information will be made publicly available and developed further by RMMS in future webinars and perspective articles.

## 1 INTRODUCTION

Severe acute respiratory syndrome coronavirus 2, SARS‐CoV‐2, led to the spread of Coronavirus Disease 2019 (COVID‐19) in December 2019 from Wuhan, China, to most of the world. COVID‐19 is a respiratory disease with wide spectrum of symptoms ranging from mild to severe and involving many different organ systems. The list of symptoms from the Centers for Disease Control and Prevention (CDC) and the World Health Organization (WHO) continues to change. As COVID‐19 is a rapidly evolving disease, clinical experience so far concludes that people aged >65 years are at a higher risk of severe illness. Underlying medical conditions, such as lung and heart diseases, immunodeficiency, obesity, diabetes, and kidney and liver diseases, increase mortality risks.^1^ Among 4103 patients in New York City between 1 March, and 2 April, 2020, hospitalization risks were greatest for those aged ≥75 years, with body mass index >40, and with heart failure.^2^ New syndromes are still being described, including skin symptoms and systemic inflammatory syndromes, such as sepsis in adults and a Kawasaki‐like syndrome in children.^3^, ^4^, ^5^

While the SARS‐CoV‐2 genome is 96% identical to bat coronavirus and shares 79.6% sequence identity to SARS‐CoV,^6^ the COVID‐19 mutant virus is believed to have a different diagnosis and prognosis from the precedent forms. SARS‐CoV‐2 has a four‐phase infection paradigm. Phase 1 includes cell invasion and viral replication through binding of the viral spike protein to a cell surface receptor called ACE2.^7^ Phase 2 includes replication of SARS‐CoV‐2 in the lung and immune system activation. In more severe cases, phase 3 includes pneumonia, and in the most severe cases, phase 4 includes acute respiratory distress syndrome, a cytokine storm, sepsis, and multiple organ failure. A cytokine storm is characterized by cascades of inflammatory cytokines being released systemiclly, including IL‐6, which has been tied to increased damage in the lungs and other organs.^8^, ^9^ Current standard of care treatment for COVID‐19 patients across these phases can be found on the CDC website, where guidelines are provided for the medical management of COVID‐19 and have been published by the National Institutes of Health (NIH).^10^

**FIGURE 1:**
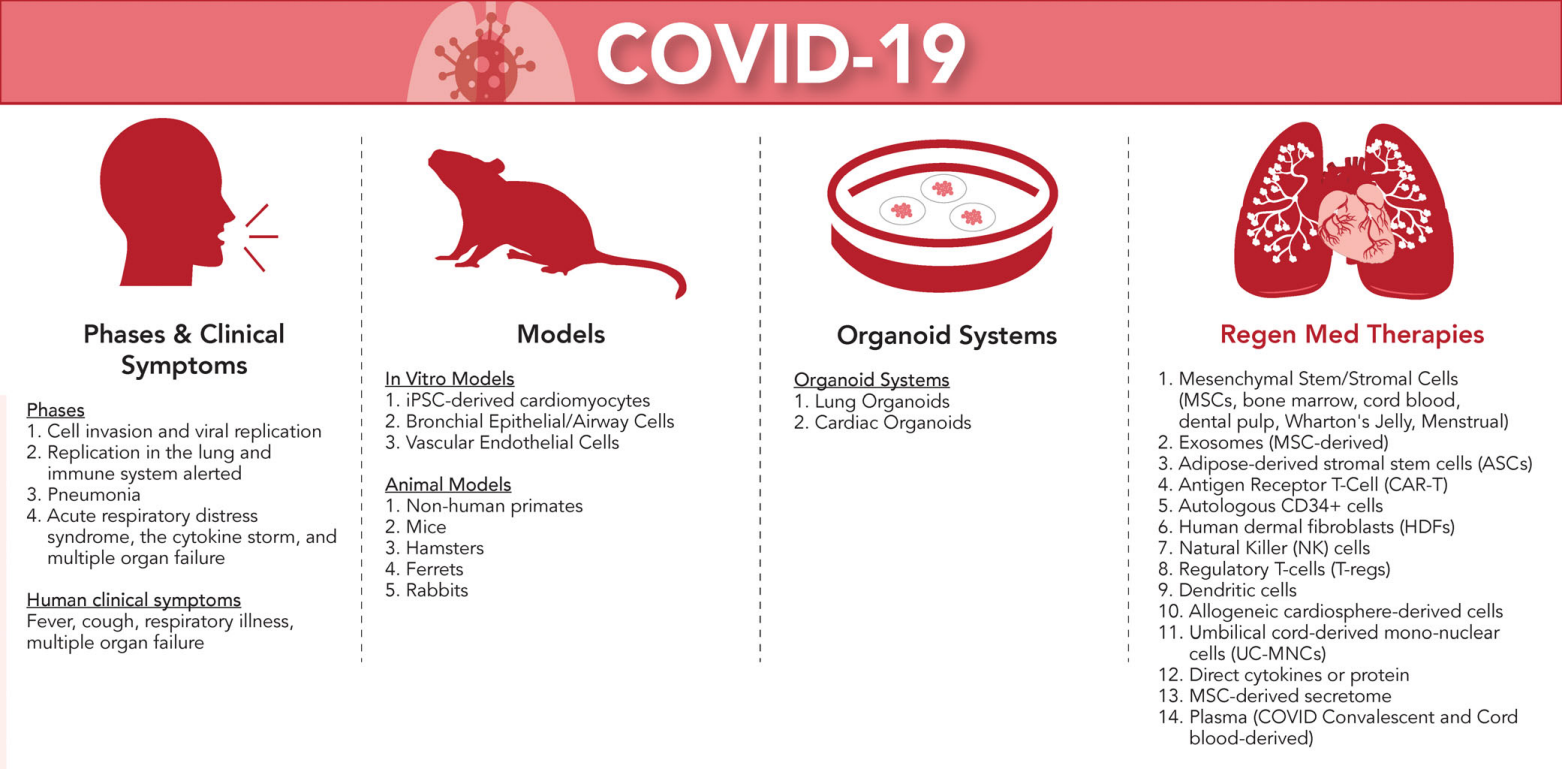
Different approaches for fighting COVID‐19. Illustrated in this graphic figure are (a) phases and clinical symptoms of COVID‐19, (b) models for COVID‐19, (c) advanced organoid systems for drug screening and personalized medicine, and (d) regenerative medicine approaches for treating COVID‐19

Regenerative medicine is uniquely poised not only to provide solid understanding of the infection mechanism and ways to prevent it, but also to introduce innovative treatments other than drugs. Figure 1 captures the phases and clinical symptoms of COVID‐19, as well as innovative ways to model and treat the disease, including organoids, immunomodulatory effects, and regenerative effects. Organoids are self‐organizing cellular three‐dimensional (3D) structures in an extracellular matrix (ECM). Organoid technology is a powerful tool for disease modeling in vitro, as they can replicate essential structural and functional aspects of organs in a dish. Alveolar organoids could improve our understanding of the antiviral inflammatory response and expedite the development of therapies.^11^ Lung organoids also offer a way to study differences in how the virus infects the lung cells of different patients and how patients might respond to tailored treatments. Other organ‐specific organoids, such as the kidney or liver, can be applied to developing therapies to prevent COVID‐19‐related organ failure.

The immunomodulatory effect of stem cell therapies has gained a rapidly growing interest during the COVID‐19 pandemic. At this writing, there are 94 clinical trials listed on ClinicalTrials.gov that are related to stem cells used for COVID‐19 therapy. Mesenchymal stromal/stem cells (MSC) have the ability to secrete a variety of endosomes and factors that reduce damaging inflammation, and several clinical trials involve administration of MSCs to COVID‐19 patients.^12^ Intravenous MSC infusion showed a reduction in inflammatory cell types and cytokines, such as tumor necrosis factor, and an increase in anti‐inflammatory cytokines, such as interleukin 10 (IL‐10). Gene expression profiles showed that MSCs were angiotensin‐converting enzyme 2‐negative (ACE2‐) and transmembrane serine protease 2‐negative (TMPRSS2‐), which indicated MSCs were resistant to COVID‐19 infection.^13^ MSCs have also been shown to assist with sepsis^14^, ^15^ and reduce inflammation.^16^

The goals of regenerative medicine—to repair, regenerate, and restore missing function or tissue—might drive the investigation of several regenerative therapies for COVID‐19‐recovered patients. In a retrospective, observational study on critically ill patients with SARS‐CoV‐2 pneumonia,^17^ the authors reported that most patients had organ function damage, including 67% with acute respiratory distress syndrome (ARDS), 29% with acute kidney injury, 29% with cardiac injury, and 29% with liver dysfunction. Moreover, type II pneumocytes in the lung are a major target of SARS‐CoV, propagating new virus and suffering widespread damage. Type II pneumocytes express ACE2 and associated enzymes transmembrane protease serine 2 (TMPRSS2) and Cathepsin L (CTSL).^18^ Lung progenitor cells (CD34 + Oct4+) can be preferentially infected by SARS‐CoV (2003 version) compared with more mature pneumocytes.^19^ Taken together, these findings mean that as COVID‐19‐infected lungs try to replace damaged and dead lung cells, the replacement stem and progenitor cells may also be targeted by the virus in a one‐two punch. This may be behind the long periods of time it takes for patients to recover, as well as the lung scarring that may reduce lung capacity, perhaps permanently for some patients. After the pandemic is past, there will be a lasting population of patients who need long‐term therapy to regain lost lung function. Any treatment that can boost the regenerative power of lung tissues by replacing these critical stem cells will help a large new patient population.

## 2 SMALL ANIMAL MODELS FOR COVID‐19

The use of the standard laboratory mouse has been limited because of the mismatch in the ACE2 receptor sequence between mouse and humans. Mice genetically engineered to carry the human ACE2 gene for research on SARS‐CoV are rapidly being assessed for their use in the study of COVID‐19. Two models, the B6.Cg‐Tg(K18‐ACE2)2Prlmn/J mice and the Tg(FOXJ1‐ACE2)1Rba mice, have been made available to researchers by public repositories. The Tg(FOXJ1‐ACE2)1Rba is from the Mouse Mutant Research and Resource, stock MMRRC:066719, and the B6.Cg‐Tg(K18‐ACE2)2Prlmn/J mouse is available from The Jackson Laboratory, JAX:034860. Their response to SARS‐CoV2 is not clear at this time.

Compared with mouse models, Syrian hamsters present more human‐like disease symptoms and pathogenesis with viruses like Ebola,^20^ and their immune responses are more similar to humans.^21^ Studies of SARS‐CoV in Syrian hamsters noted viral replication in the lungs with clearance in 7 days; however, the hamsters did not progress to ARDS.^22^ While Syrian hamsters clearly have their niche in infectious diseases, there are also limitations to their use. Species‐specific reagents such as antibodies are scarce. Still, while the Syrian hamster is a promising model for the study of COVID‐19, their limited commercial availability and widespread use present practical limitations in their high throughput use.

Ferrets are good models for respiratory diseases, as their lung and airway physiology are close to humans. Unlike rodents, ferrets cough and sneeze, making them a useful model of disease transmission. When infected with SARS‐CoV‐2, ferrets have elevated temperatures, lethargy, and appetite loss but do not progress to ARDS. There are also few research facilities capable of working with ferrets, which have complicated animal husbandry requirements and a limited commercial supply.^23^

Each animal model offers advantages that will be useful, not only for the testing of therapies, but also for understanding related co‐morbidities. Interested readers are referred to this excellent reference, which compares 10 different animal models in the search for the best model to study COVID‐19.^24^

## 3 ADVANCED ORGANOID SYSTEMS FOR MODELING COVID‐19

Organoids can model organ function in‐vitro, providing a more accessible, faster, and higher throughput screening tool than in vivo models. There are a few methods to engineer organoids, including manual 3D culture, liquid handling, or 3D bioprinting. All of these methods can use ECM in the process, which results in cell assembly that recapitulates organ function on a small scale and can help model different clinical features of COVID‐19.^25^ Using organoids to model organ function and infection by the SARS‐CoV‐2 virus can be a valuable tool to gather more insights on its viral tropism and pathogenesis, as well as serve as a platform to find potential treatment strategies for COVID‐19. While initial studies with SARS‐CoV‐2 have used Vero E6 cells, using models of the lung, heart, kidney, and intestine can be crucial to new therapies.

Lung organoids have previously been used to model infections by different pathogens. Hui et al. developed human lung organoids that recapitulate the bronchial airway, including cilia, goblet cells, club cells, and basal cells.^26^ They then successfully infected these organoids with the influenza virus, analyzing replication competence, tissue tropism, and host responses. Their results were comparable to those observed in human ex vivo bronchus cultures, offering a viable alternative for modeling viral infections in the lung.

Similarly, Monteil et al used SARS‐CoV‐2‐infected human kidney organoids to investigate the effectiveness of human recombinant soluble ACE2 (hrsACE2) in inhibiting viral growth.^27^ Liver organoids were also utilized to investigate how this tissue is infected and damaged by SARS‐CoV‐2. Zhao et al found evidence to support the hypothesis that COVID‐19 patient liver damage might be resultant from cholangiocyte injury and consequent bile acid accumulation caused by the viral infection.^28^ These results indicate that organoids are a powerful tool in understanding mechanisms through which SARS‐CoV‐2 affects liver tissue.

Although cardiac organoid infection by the SARS‐CoV‐2 virus has not been characterized, cardiac organoids could be a feasible platform for use in COVID‐19 drug screening. Mills et al, for example, developed a high‐throughput bioengineered human cardiac organoid platform, using it to screen 105 small molecules with regenerative potential.^29^ These organoids could also be used to study how the heart is affected by COVID‐19.

The intestine is another target organ for the SARS‐CoV‐2 virus, with a high expression of ACE2 in intestinal enterocytes. Lamers et al. developed human small intestinal organoids (hSIOs) that were readily infected by SARS‐CoV and SARS‐CoV‐2 viruses.^30^ Their study indicated enterocytes as the target cell for infection in the intestine, demonstrating that intestinal organoid cultures can be a key method for better understanding viral biology.

Immune organoid culture can also contribute to understanding COVID‐19 immune responses.^31^ Organoids are a useful tool to investigate and characterize organ function and disease progression, and they should be further explored as a high‐throughput, highly specific platform for COVID‐19 drug screening.

**FIGURE 2:**
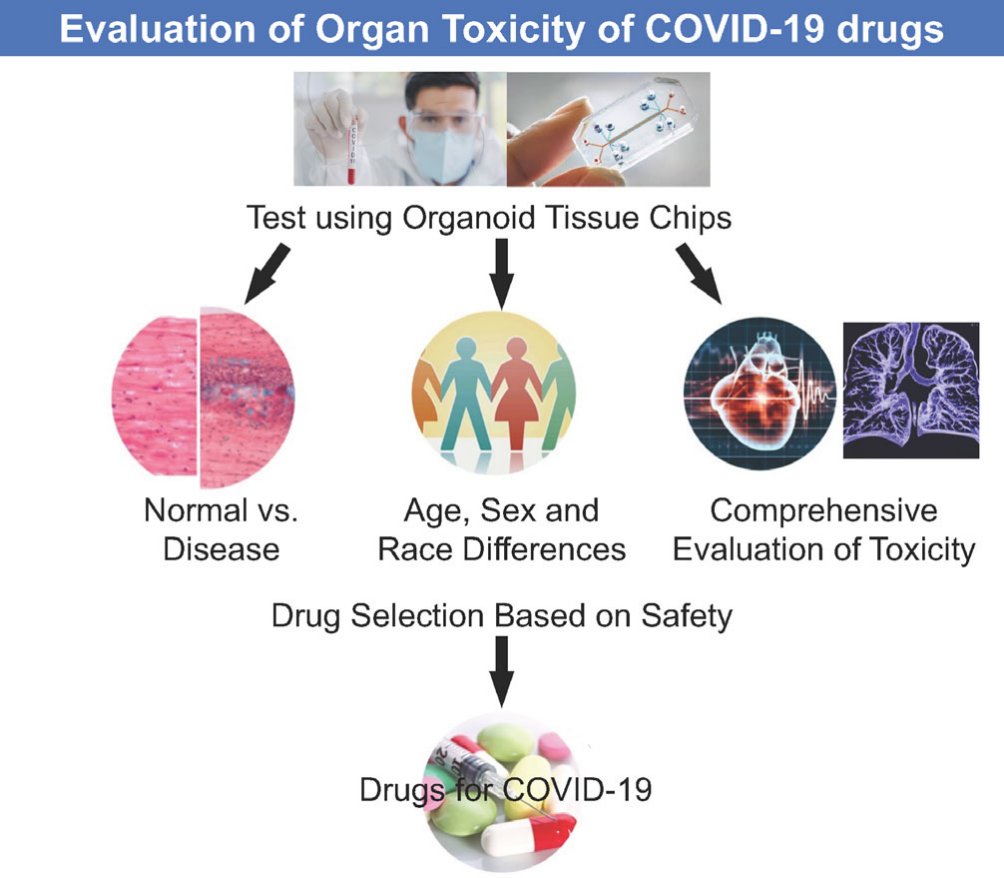
Advanced organoid tissue chips for evaluating organ toxicity of COVID‐19 drugs. This figure shows how testing could be performed using organoid tissue chips to screen potential toxicity in drug candidates

Human in vitro tissue‐on‐a‐chip platforms enable the direct use of human heart cells to evaluate potential effectiveness of existing or new COVID‐19 drugs (Figure 2). Additionally, using cells from a diverse patient population, including both sexes, may help classify the cardiovascular and pulmonary risk of COVID‐19 drugs. Additionally, there is need to screen repurposed drugs for COVID‐19 for toxicity, and an excellent review has evaluated current therapeutic drugs for the treatment of COVID‐19 patients.^32^

Tissue chips using lung airway epithelium that express high levels of ACE2 and TMPRSS2 have been used to screen repurposed drug candidates for inhibition of SARS‐CoV‐2 viral entry.^27^ When used to assess seven clinically approved drugs (chloroquine, arbidol, toremifene, clomiphene, amodiaquine, verapamil, and amiodarone), only toremifene and amodiaquine were found to inhibit viral entry. These results suggested that human tissue chip technology screening assays could be used to study human disease pathogens and expedite drug repurposing.^33^ Figure 2 highlights how organ toxicity of COVID‐19 drugs could be evaluated using these organoid tissue chips, where advances in connecting these organ chips to achieve multi‐tissue interactions will be important to detect unanticipated drug toxicity.^34^ Taken together, employing an advanced organoid tissue chip can reduce the cost and time needed to develop new treatments, due to the ability to test so many compounds across these different organ systems, which could be created for thousands and thousands of patients. This idea is in fact being taken to a new level by the NIH, which has deployed funding opportunities for clinical trials on a chip. The future will harness these tissue chips and use large data repositories to essentially perform clinical trials using patient‐derived organoid models.

## 4 REGEN MED THERAPIES FOR COVID‐19

Patients suffering from severe COVID‐19 have increased proinflammatory cytokine levels, including increased IL‐6 levels that can be part of the cytokine storm or cytokine release syndrome (CRS).^35^ CRS is not new to the regenerative medicine industry. Chimeric antigen receptor T‐cell therapy (CAR‐T), the first FDA‐approved cell therapy, can elicit CRS. Since the beginning of the current pandemic, the regenerative medicine industry has rapidly pivoted to translate the lessons learned from mitigating CRS in CAR‐T therapy to COVID‐19.^36^ Recent results from the Randomized Evaluation of COVID‐19 Therapy (RECOVERY) trial (ClinicalTrials.gov Identifier: NCT04381936) confirmed that patients hospitalized with COVID‐19 receiving dexamethasone (a corticosteroid medication) resulted in lower 28‐day mortality among those receiving either mechanical ventilation or oxygen alone.^37^ This study supports the tremendous benefits of large randomized studies in evaluating interventions for COVID‐19. Previous clinical data did not support the use of corticosteroid treatment for COVID‐19‐related lung injury.^38^ Therefore, there is an emergent need for more large randomized trials, such as RECOVERY, to develop new treatments for COVID‐19‐associated CRS and ARDS.

MSCs are the leading potential allogeneic cellular therapy candidate for ARDS and CRS.^39^ Once at the site of infection, MSCs release anti‐inflammatory cytokines and act through cell‐cell contact to help regulate the local environment. Early studies in China have demonstrated the efficacy of MSCs in COVID‐19 patients and some have already been published.^13^ Outside of China, promising results in small cohorts have already shown some success and been announced. Pluristem Therapeutics showed that infusion of placenta‐derived “mesenchymal‐like cells” in COVID‐19 patients with ARDS on mechanical ventilation led to improvements in four of six patients.^40^ Under emergency use, RESTEM recently showed promising results from infusion with experimental umbilical cord lining‐derived stem cells in three COVID‐19 patients with ARDS. On the basis of these results, the RESTEM has received FDA approval for a 60‐patient phase I/II clinical trial.^41^ The University of Miami Medical School also has FDA approval to test cryopreserved allogenic umbilical cord stem cells on 12 COVID‐19 patients.^42^ In terms of the allogeneic approach with bone‐marrow derived MSCs, Remestemcel‐L, developed by Mesoblast, has an encouraging potential after 9 of 12 moderate to severe COVID‐19 patients successfully came off ventilators within 10 days of two infusions.^43^ Athersys also released positive 28‐day data with 1‐year follow‐up on an ARDS trial using MultiStem, which showed improvement in survival and improvements in ventilator dependency. As a result, the MultiStem has been given fast‐track designation by the FDA, and MACOVIA, a pivotal phase II/III trial will enroll ~400 COVID‐19 patients. The FDA also recently approved a phase II single‐arm study for Hope Biosciences (Houston, TX) to enroll 75 high‐risk participants using autologous, adipose‐derived MSCs.^44^ In addition to these trials, more than 200 additional MSC‐related clinical trials for COVID‐19 have been registered on ClinicalTrials.gov as of August 10, 2020. This fairly extensive clinical effort to develop an MSC therapy includes sources from umbilical cord, bone marrow, adipose tissue, and dental pulp. Leveraging some early positive results, more extensive well‐designed clinical trials are ongoing, which will be critical in establishing the effects of MSC treatments on COVID‐19 patient survival and accelerated recovery.

Regulatory T cell (Treg) transfusion may also be efficacious against COVID‐19 associated CRS. Like MSCs, Tregs reduce immune responses and play a role in the prevention of autoimmunity, allergy, organ transplant rejection, and other inflammatory immune responses.^45^ Allogeneic Treg therapy has been explored as a treatment for amyotrophic lateral sclerosis. Recently, Cellenkos^46^ submitted a clinical development proposal to the Biomedical Advanced Research and Development Authority (BARDA) for phase I/II clinical trials for COVID‐19 using cord blood Tregs.

Capricor Therapeutics recently announced that treatment with allogeneic cardiosphere‐derived cells led to four of six COVID‐19 patients being removed from mechanical ventilation within 1‐4 days post‐transfusion. As a result of this success, the US FDA has approved the company’s expanded access protocol allowing for treatment of twenty additional COVID‐19 patients.^47^ While early results are very promising, these preliminary reports lack proper control arms and placebos, and further trials are necessary.

Rather than quashing immune responses, boosting immune responses using natural killer (NK) cells also has been proposed as a treatment for severe COVID‐19. Celularity Inc. recently received FDA approval to initiate a trial in 86 COVID‐19 patients. Celularity’s placental stem cell‐derived NK cells may be able to target and eliminate virally infected cells; however, there are risks that NK cells may add to COVID‐related inflammation and that they might not be able to detect which cells are infected.^48^

Overall, regenerative medicine offers tremendous hope for treating COVID‐19, and initial clinical trial results are promising. However, properly designed and conducted clinical trials, with appropriate enrollment, controls, and evaluation, are still needed.

## 5 TECHNOLOGIES AND RESOURCES TO FIGHT COVID‐19

With great challenges come great opportunities for the scientific community. Several resources have been made available to combat COVID‐19 over a short period of time since the deceleration of the pandemic. In the future, we envision doing more with less, fully automating standardized workflows, using artificial intelligence (AI) to mine data and develop learning and predictive algorithms and using modular GMP environments to optimize manufacturing processes.

Social distancing requirements will be imposed on laboratories in response to the COVID‐19 pandemic, so automation will improve the standard workflow in the laboratory and minimize the use of people and personal protective equipment. Recent publications discuss automation and standardization of processing for a potential miRNA marker candidates.^49^, ^50^ For instance, the Revos Tissue Processor (Thermo‐Fisher) and high resolution slide scanners, such as those from 3DHISTECH, reduce labor time and maintain efficiency during reduced staff schedules. It will also be advantageous to pair these technologies with archiving solutions like the ARCOS platform from EPREDIA for efficient data storage and recovery and potentially to be combined with machine learning and predictive algorithms. With remote access to images, research can continue even during limited access to a facility. Building upon this, the adoption of cloud‐based AI solutions further enables remote access to data. One such ecosystem, a spin‐off from Memorial Sloan Kettering Hospital, has assisted in accelerating biomarker discovery by PAIGE.AI.^51^

Cell therapies production models are moving toward closed systems to reduce contamination risks. Closed bioreactors are gaining traction for cell expansions steps. Closed, modular cGMP cell production environments that can enclose all the cell production steps from start to finish are available and will be implemented in a post‐COVID‐19 world. These systems have flexibility to be configured in non‐GMP space and validated in weeks. The clonability of the system means that once conditions are optimized for production of the most potent cell product (O_2_, CO_2_, temperature, humidity), those same conditions can be verifiably reproduced anywhere in the world. This adds scientific reproducibility and reliability to distributed COVID‐19 organoid and cell therapy production models.

A new potential resource is the use of commercially available small satellites in low Earth orbit to be used as a microgravity research platform, in conjunction with tissue chip/lab‐on‐a‐chip technology and/or microfluidic devices such as what the National Center for Advancing Translational Science is working on in collaboration with the Defense Advanced Research Projects Agency and the Food and Drug Administration.^52^ Companies and research institutions are finding considerable value in exploring the effects of the space environment on human health, pharmaceuticals, biotechnology/bioengineering, and advanced manufacturing. In fact, studies have shown that microgravity can cause various pathogens to become more virulent,^53^ which has a genetic component,^54^ and this could be used to develop accelerated disease progression models in organoids infected with the SARS‐CoV‐2 virus. The space environment has properties not found in terrestrial environments, which includes microgravity, hard vacuum, and space radiation. These properties have surprising effects on cellular and molecular processes. Opportunities exist for elucidating molecular mechanisms in microgravity, studying complex fluid dynamics, testing materials, analyzing proteins and large molecules, and advancing the science of nanofluidics and biotechnology. For example, Orbital Transports, Inc., is developing solutions for performing cellular and molecular research on small satellites by using a suitably sized bioreactor, tissue chip/lab‐on‐a‐chip technology, and/or microfluidic devices. This will enable new opportunities for space‐based research that can be conducted without human intervention, and thus, free the scientist from crew time and other limitations inherent to the mission of the International Space Station.

The world’s leading journals are providing free access to COVID‐19‐related papers and resources. *The New England Journal of Medicine* has a COVID‐19 landing page that compiles all of their COVID‐19 content.^55^ *The Lancet* also has a resource center focused on COVID‐19 and provides free access.^56^ The journal *Nature*’s newsletter, *Nature Briefing*, is a weekly gathering of the latest information.^57^ COVID‐specific literature web search engines, such as COVID Scholar,^58^ have also appeared. Utilizing these free reliable resources will give you the latest, substantiated scientific developments under way to combat COVID‐19.

The US FDA has done an outstanding job in response to COVID‐19. The FDA has fast‐tracked potential treatments (~60 day review) and has been very responsive in approving Emergency Use Authorizations. They also have a dedicated specific resources: Coronavirus Disease 2019 (COVID‐19),^59^ Emergency Use Authorizations,^60^ and Coronavirus Treatment Acceleration Program (CTAP).^61^ Additional resources to get the complete picture of the developments taking place are also available at the following organizations: World Health Organization (WHO),^62^ Centers for Disease Control and Prevention (CDC),^63^ John Hopkins,^64^ Chinese CDC,^65^ and Gates Notes.^66^ *The Atlantic* has developed The COVID Tracking Project^67^ to focus on the incredibly important topic of testing and compiles the most accurate data available. Eva Garland Consulting LLC has done a great job of compiling and continuously updating the grants opportunities.^68^ CellTrials.org also provides a free report on all registered COVID‐19 trials.^69^

COVID‐evidence^70^ is a non‐profit initiative of the Department of Clinical Research at University of Basel and the Meta‐Research Innovation Center at Stanford, with other international partners and collaborators, which provides a continuously updated free database on interventions for COVID‐19. BioCentury has a COVID‐19 Resource Center^71^ with an information portal on COVID‐19 therapies and vaccines (clinical and preclinical), COVID‐19 trial timeline, diagnostics, status of regulatory meetings, resources for researchers, and a COVID‐19 research gateway where companies and researchers are provided an intake form to submit their latest advances on compounds, diagnostics, preclinical assays, clinical trial designs, protocols, sites, data or data‐sharing resources, and inquiries about COVID research and development.

The Regenerative Medicine Manufacturing Society (RMMS) and *STEM CELLS Translational Medicine* (SCTM) have created a page on the *STEM CELLS* Portal for regenerative medicine resources in the fight against COVID‐19. This page provides a COVID‐19 Resource Submission Page^72^ for scientists (academic, industry, government, non‐profits, etc.) to highlight regenerative medicine resources directed against COVID‐19 in three areas: (a) models, (b) cell therapies, and (c) technologies. These resources will be presented in a redacted format (type of regenerative medicine resource / institution / resource description) on the Regenerative Medicine COVID‐19 Resources Results Page^73^ and shared with RMMS working group co‐leads to continue meaningful discussions within the working groups and also to plan future webinars and perspective articles to disseminate these important regenerative medicine resources to the entire scientific community. Those wishing to become involved in RMMS working groups are encouraged to visit our website (http://regenmedmanufacturing.org/membership/) and sign up to be a member.

The scientific community continues to work diligently and cooperatively to combat COVID‐19 and the coronavirus. Through collaboration, reliance on real‐world evidence, and a willingness to roll up our sleeves and get to work, we will overcome this pandemic together.

## 6 CONCLUSIONS AND FUTURE DIRECTIONS

Regenerative medicine is poised to make a true difference in the fight against COVID‐19. We have reviewed some of the advances in organoid systems to model COVID‐19 symptoms that could be used for developing new treatments or screening at‐risk patients. We have also covered some of the effects regenerative medicine‐based therapies may have on treating COVID‐19‐related symptoms, such as immunomodulatory effects or even regenerative effects. We have discussed technologies and resources that are currently available to confront COVID‐19. It is times like these when we must all come together, share our talents and resources, and develop the best methods, technologies, and capabilities to address global health challenges.

## CONFLICT OF INTEREST

A.D.H. declared leadership position BioSpherix, Ltd. J.K. declared consultant/advisory role with Orbital Transports, Inc. S.L. declared employment with PHC Corporation. K.C. declared employment and stock ownership with Auxocell Laboratories, Inc. M.A. declared employment and stock ownership with Humabiologics, Inc. S.E. declared employment with Cytiva. The other authors declared no potential conflicts of interest.

## AUTHOR CONTRIBUTIONS

A.A., M.L., T.P.: critical review and revision of the advanced organoid systems for modeling section of the manuscript; A.H., M.A.: conception and design, collection and/or assembly of the data, critical review and revision of the manuscript; T.A., J.G.: critical review and revision of the Regen Med Therapies for COVID‐19 section of the manuscript; J.K., S.L., K.C.: critical review and revision of the technologies and resources section; C.L.: critical review and revision of the small animal models section of the manuscript; S.E., J.H.: conception and design, collection and/or assembly of the data, manuscript writing, final approval of manuscript.
